# Preface to Special Topic on Protein Dynamics: Beyond Static Snapshots in Structural Biology

**DOI:** 10.1063/1.4942424

**Published:** 2016-02-26

**Authors:** George N. Phillips, José N. Onuchic

**Affiliations:** 1 Department of BioSciences and Chemistry, Rice University, Houston, Texas 77005, USA; 2 Center for Theoretical Biological Physics and Department of Physics, Rice University, Houston, Texas 77005, USA

As structural biology moves beyond the over dependence on the static snapshots to a deeper understanding of the role of dynamics in the function of the molecules, it has never been more apparent that experiment, theory, and simulation must be all considered together in our discourse. This special issue of *Structural Dynamics* is devoted to the subject of protein dynamics and comprises articles that span these areas nicely and show how the combined information coming from them has completely changed the field. The papers include descriptions of new experimental crystallographic evidence on the flexibility of loop dynamics in an enzyme involved in natural product biosynthesis (Han *et al.*, 2016); the effects of quaternary structure on the dynamics of pilin as measured by HD exchange methods (Lento *et al.*, 2016) and as predicted by simulations in hemoglobins (Gupta and Meuwly, 2016); efficient methods for constraining atomic level molecular dynamics trajectories (Chandrasekaran *et al.*, 2016), theory and simulations of assembly of complicated fibrous structures in muscle contraction (Fischer *et al.*, 2016); and theoretical methods for defining sections of proteins that form fairly rigid segments as a dimensionality reduction tool (Streinu, 2016).

Taken together these articles set an example of how multidisciplinary thinking is fundamental in modern structural biology. The home departments of the authors include biosciences, chemistry, physics, and mathematics, each bringing a unique perspective yet addressing the same topic—how the movements of the atoms in time are connected to potentially satisfying mechanistic understandings of biological phenomena. Furthermore, all the discussion can be distilled to a common concept; that of an energy landscape, in which the proteins roam from place to place under thermal or other sources of energy to traverse boundaries.

Biophysical techniques for observing, calculating, or describing the flexible nature of proteins and their resulting ensembles continue to improve in both speed and accuracy. Cryoelectron microscopy, X-ray diffraction, and magnetic resonance methods all promise to deliver single-molecule level structure determinations, by building a structure from many noisy snapshots, using “diffract and destroy” free electron laser illumination or by nanoscale magnetic resonance imaging. If these new ideas come to fruition, scientists would be able to look at individual examples of conformations and at the ensembles directly. This experimental advance, coupled with powerful theory and computational methods, would really accelerate our explorations of the molecular world of protein dynamics.
